# Esophageal, Gastric, and Duodenal Histologic Findings in Patients with Feeding Difficulties

**DOI:** 10.3390/nu12092822

**Published:** 2020-09-15

**Authors:** Jensen Edwards, Craig Friesen, Amy Issa, Sarah Edwards

**Affiliations:** 1 Children’s Mercy Kansas City, Kansas City, MO 64108, USA; jensen.edwards@ttuhsc.edu (J.E.); cfriesen@cmh.edu (C.F.); aissa@cmh.edu (A.I.); 2 School of Medicine, Texas Tech University Health Sciences Center El Paso, El Paso, TX 79905, USA; 3 School of Medicine, University of Missouri at Kansas City, Kansas City, MO 64108, USA

**Keywords:** feeding, evaluation, EGD, gastrointestinal, pathology

## Abstract

Currently, there are inconsistencies in the recommendations of when to obtain an esophagogastroduodenoscopy (EGD) in children with feeding difficulties. The aim of our study was to identify EGD findings in patients presenting to a large, outpatient feeding program. Additionally, we investigated the presence of any relationship between abnormal pathology seen on biopsies (inflammation) and symptoms of feeding intolerance such as vomiting, gagging, retching, or abdominal pain. Retrospective analysis of electronic medical records (EMRs) was conducted for all new patients aged 0–17 years presenting to the Multidisciplinary Feeding Clinic. Three hundred and thirty patients (50.2%) had an EGD with complete biopsies. Of these 330 patients, biopsies revealed esophagitis in 40%, gastritis in 33.6%, and duodenitis in 15.2%. Overall, 61.21% had an abnormal pathology in at least one site. We found that children with feeding disorders commonly have esophagitis, gastritis, and/or duodenitis and that symptoms are poor predictors of pathology. This study underscores the importance of gastrointestinal evaluation as part of a multidisciplinary evaluation in patients with feeding difficulties.

## 1. Introduction

Feeding difficulties (FDs) are present in 50% of children with normal development and 80% of children with developmental problems, highlighting the prevalence of these conditions and the need for effective diagnostics and treatment [1]. Historically, FDs have been classified into one of two dichotomies: organic or non-organic. However, this classification system is not all-encompassing as more than one system is often disrupted. To perform a complete workup, Goday et al. recommended that FDs require a comprehensive assessment due to the involvement of four domains: medical, nutritional, feeding skill, and psychosocial dysfunction [2].

A typical evaluation of children with FD involves a multidisciplinary approach, with occupational and speech therapists, dietitians, social workers, and pediatric specialists all playing various roles [1]. The first step of the feeding assessment should be to present a comprehensive history. This history includes the caregiver’s perception of the child’s feeding difficulties, the child’s growth and development, current diet, and a description of the typical feeding environment [3]. Medical and social issues can also have a profound impact on feeding and should be reviewed when recording a patient’s history [3]. Once a thorough history is documented, the next steps include an observed feeding and a complete physical examination. This comprehensive clinical assessment defines the need for more invasive studies. These can include laboratory studies, radiographic studies, or endoscopic procedures depending on the symptoms at presentation. Current literature recommends proceeding with an esophagogastroduodenoscopy (EGD) when there are additional symptoms, but there is no precedent for children with feeding disorders alone [4].

The etiology of feeding difficulties in children is often multifactorial, including medical, oral motor, and behavioral etiologies [5]. Studies have indicated gastrointestinal (GI) etiologies as the primary medical component in children with feeding problems. Rivera et al. identified red flags present in children with FDs that indicate an underlying GI pathology. These red flag symptoms include dysphagia, aspiration, odynophagia, coughing, choking, pneumonia, vomiting, diarrhea, failure to thrive, autism, and congenital/developmental anomalies [1]. Similar to the concept of red flag symptoms, Reedy et al. identified various alarm symptoms that would warrant an EGD. In the presence of alarm symptoms, Reedy et al. noted that EGD displays high diagnostic yield when evaluating children with abdominal pain [4]. However, in the absence of alarm symptoms, EGD diagnostic yield has not yet been established.

Gastrointestinal pathology has the potential to significantly alter feeding. Sdravou et al. noted that children with GI conditions may develop a restricted diet, practice selectivity, feeding refusal, and acquire general anxiety over feeding [6]. For instance, Mukkada et al. conducted a retrospective study involving 200 children from a multidisciplinary eosinophilic diseases program, where 33 children from the cohort also had FDs [7]. These dysfunctional behaviors may lead to reduced dietary intake, which can negatively impact growth and development and may persist even after treating the disease or withdrawing the allergenic stimulus. Once specific feeding behaviors are learned, abnormal motor patterns are difficult to unlearn.

There is a pressing need for early detection and appropriate individualized treatment of GI medical conditions to eliminate the development of associated feeding problems and their consequences, both short- and long-term. Currently, there are inconsistencies with recommendations on when to obtain an EGD in children with feeding difficulties. The aim of our study was to identify EGD findings in patients presenting to a large, outpatient feeding program. Additionally, we investigated the presence of any relationship between abnormal pathology and symptoms of feeding intolerance such as vomiting, gagging, retching, or abdominal pain.

## 2. Materials and Methods

We performed a single-center, retrospective analysis of electronic medical records (EMRs) for all new patients aged 0–17 years presenting to the Multidisciplinary Feeding Clinic at Children’s Mercy Hospital between 1 October, 2014 and 31 December, 2019. Medical records were reviewed for sex, age at the time of referral, clinic notes, symptoms, presence of a feeding tube, and endoscopic findings including histology, which were entered into a restricted, secure database. Children’s Mercy Kansas City (CMKC) Institutional Review Board approved the study.

All patients were further categorized by symptoms on the initial clinic visit. The symptoms analyzed were dysphagia, gagging, retching, vomiting, choking, abdominal pain, limited variety of food, limited volume of food, feeding refusal, coughing, regurgitation, wheezing, globus sensation, nausea, and ingesting non-food items. Symptoms were based on parent or patient report and were chosen based on standard questions asked at each Multidisciplinary Feeding Clinic visit. Due to the inability to report symptoms such as nausea and globus sensation across age groups and developmental status, these symptoms were removed. Feeding tube status was also analyzed and further categorized into gastric (G), nasogastric (NG), nasojejunal (NJ), and gastrojejunal (GJ) tubes.

All biopsy assessments were performed by board-certified pediatric pathologists as part of routine clinical care. Esophagitis was defined as gastroesophageal reflux disease when peak esophageal eosinophil counts were less than 15 per high-powered field and as eosinophilic esophagitis when 15 or greater. Gastritis and duodenitis were defined as chronic when only lymphocytes and/or plasma cells were elevated, as active if neutrophils were present, and as eosinophilic if increased eosinophils were noted in the lamina propria. The presence of *Helicobacter pylori* on gastric histology was recorded.

All statistical analyses were performed with SPSS version 23 (SPSS, Inc.; Chicago, IL, USA). Categorical variables (e.g., presence of individual symptoms, presence of inflammation) were compared using the Chi-squared test or the Fischer’s exact test, whichever was appropriate. Continuous variables (e.g., age) were compared using Student’s *t*-test. A *p*-value <0.05 was considered significant.

## 3. Results

Six hundred and sixty-three patients were seen for evaluation in the Multidisciplinary Feeding Clinic during this time frame. Five patients were removed from the study: one patient did not present to the clinic appointment, one was not seen in the Multidisciplinary Feeding Clinic, and three were found to have duplicate medical record numbers. Of the 658 patients included in the study, 336 had an EGD. We identified patients who had an EGD with biopsies of the esophagus, gastric antrum, and duodenum. Six patients were excluded from statistical analysis of pathology due to having incomplete biopsies, leaving 330 patients with complete sets of biopsies. The majority (64.3%) of patients were male. There was no significant difference (*p* = 0.615) in sex between patients with an EGD (36% female) and patients without (35% female). There was also no significant difference (*p* = 0.378) in age between patients with an EGD (4.4 ± 3.5 years) and patients without (4.5 ± 3.6 years). Three symptoms were significantly increased in patients with an EGD: vomiting, coughing, and choking. Of note, abdominal pain was trending toward significance (*p* = 0.056). The frequencies of specific symptoms between patients with an EGD vs. those without are listed in Table 1.

Three hundred and thirty patients (50.2%) had an EGD with complete biopsies. Of these, biopsies revealed esophagitis in 40%, gastritis in 33.6%, and duodenitis in 15.2%. Overall, 61.21% had an abnormal pathology in at least one site. The frequencies of specific biopsy findings can be found in Table 2. Of note, biopsies of two patients indicated Candida esophagitis; one had inflammation on an esophageal biopsy, and one had an otherwise normal esophageal biopsy, i.e., non-invasive. In addition, of the patients with chronic duodenitis, three had a confirmed diagnosis of celiac disease and two had possible celiac disease, which is defined as a biopsy consistent with celiac but no celiac antibody testing.

Of the 330 patients with complete upper endoscopy biopsies, 25.6% had a G-tube, 5.4% had a NG tube, 0.3% had a NJ tube, and 1.5% had a GJ tube. There was no significant difference in the frequency of esophagitis or gastritis comparing patients with a feeding tube to those without a feeding tube. There was a trend toward increased duodenitis (*p* = 0.058) in patients with a feeding tube. The specific frequencies of biopsy results in patients with and without a feeding tube can be found in Table 3.

We further investigated the relationship between symptoms and EGD biopsy results. In patients with esophagitis, only the symptom of a limited variety of food was trending toward a significant increase, as shown in Table 4. In patients without gastritis, there were two symptoms with increased frequencies: feeding refusal and gagging. The specific symptom frequencies in patients with and without gastritis are provided in Table 5. Lastly, in patients with duodenitis, only the symptom of feeding refusal was trending toward increased significance, as shown in Table 6.

## 4. Discussion

The current findings underscored that abnormal EGD biopsy findings are common in children with FD, as indicated by 40% of patients with esophagitis, 33.6% with gastritis, and 15.2% with duodenitis. Rommel et al. reported the etiologies of feeding difficulties in their large outpatient feeding program. They reported reflux esophagitis in 60% of their patients, but it is unclear if their diagnoses were clinical, mucosal, or a combination [5]. Rivera et al. evaluated 93 patients with feeding difficulties, of which EGDs were performed in 67 patients, although not all patients had a full set of biopsies [1]. When biopsies were performed, they found esophagitis in 53%, chronic gastritis in 26%, and abnormal small bowel biopsies in 6.5% [1]. Eosinophilic esophagitis (EoE) was present in 24% of patients where esophageal biopsies were obtained, which is somewhat higher than observed in the current study. This could be due to the differences in sample sizes, patient heterogeneity, or differences in patient selection for endoscopy.

Subtyping EGD biopsy findings of inflammation into categories such as histologic esophagitis, chronic gastritis, and eosinophilia is an addition to previous feeding difficulty literature. The most common etiology of esophagitis in our patient population was reflux esophagitis (72.7%). Reflux esophagitis can often lead to erosive esophagitis, which is prevalent in 1% of the population [8]. Subtyping esophagitis can allow different treatment options, as reflux esophagitis responds to proton pump inhibitors, whereas eosinophilic esophagitis may also require topical steroids or diet restrictions [8]. For gastritis, the most common etiology was chronic gastritis (60.4%). Chronic gastritis may predispose to increased gastric sensitivity, which could alter both the variety and the volume of food intake [9,10]. For duodenal biopsies, eosinophilia (35%) was the most prevalent cause of inflammation. Duodenal eosinophilia may be idiopathic or secondary to food allergy, complementing the notion that children with food allergies often have feeding difficulty [7,11].

Next, we assessed relationships between symptoms and biopsy results. There were no symptoms significantly associated with esophagitis or duodenitis. There was a trend towards limited variety of food being associated with increased esophagitis prevalence and feeding refusal being associated with increased duodenitis prevalence. These two symptoms are not recognized as alarm or red flag symptoms. When assessing histology of the gastric antrum, there were two symptoms that were negative predictors of gastritis: feeding refusal and gagging. Currently, symptoms are the main indicators for performing endoscopy, so we thought it was important to assess for symptom bias in the selection of patients in the current study. There were three symptoms that increased in those who had an EGD compared to those who did not have an EGD. These symptoms were vomiting, coughing, and choking, with abdominal pain trending toward significance. These symptoms fall under the category of previously-recognized red flag symptoms that are associated with underlying GI pathology according to Rivera et al. [1]. Only vomiting falls under the category of alarm symptoms associated with high EGD diagnostic yield according to Reedy et al. [4]. Although these differences were statistically significant, they were modest in magnitude and unlikely to be clinically significant. Our study indicated that symptoms lack the ability to predict the presence of mucosal pathology. Even symptoms shown to be statistically significant between patients with and without inflammation had a poor ability to predict histologic findings.

We also investigated whether or not patients with feeding tubes may be more likely to have abnormal biopsy findings, in part to determine if the need for a tube indicates pathology, but also to assess for the potential of the tube to cause inflammation. There were no significant differences in esophagitis, gastritis, or duodenitis between patients with and without tubes, indicating that the presence of a feeding tube alone would not be a good predictor of mucosal pathology in our population.

We recognize our study has several limitations given the retrospective nature. These include the absence of comorbidities to better understand the medical complexity of those patients who underwent endoscopy compared to those who did not. There was also the possibility of bias in the selection of patients for endoscopy. We found small but clinically significant increases in frequencies for vomiting, choking, and coughing in those patients who underwent endoscopy. It is also possible that there was variability with regard to how symptoms are reported by parents, but this represents a real-world clinical reality inherent in the care of these patients. Lastly, given the ethical prohibition on performing endoscopy on healthy children to obtain control data, we were unable to determine if the rates of inflammation were greater than in controls. The scope of the current study, however, was limited to describing the prevalence of the various histologic findings in this patient group. Future studies should assess the clinical importance of the histologic findings in predicting treatment response in children with FD.

## 5. Conclusions

We showed that children with FD commonly have esophagitis, gastritis, and/or duodenitis and that symptoms are poor predictors of pathology. This suggested that FD is a predictor of pathology, independent of symptoms that have usually been viewed as indications for endoscopy. The findings underscore the potential importance of gastrointestinal evaluation by a pediatric gastroenterologist as part of the multidisciplinary evaluation of patients with feeding difficulties. Ultimately, further studies are needed to determine the significance of the various forms of inflammation to understand whether they have value in predicting prognosis or, more importantly, whether they are therapeutic targets, which, when sufficiently treated, may result in feeding improvement.

## Figures and Tables

**Table 1:** Symptom frequencies of patients with an EGD vs. those without an EGD.

Symptom	EGD	No EGD	*p*-Value
Dysphagia	23%	25%	0.625
Gagging	33%	30%	0.470
Retching	0.3%	2%	0.090
Vomiting	36%	22%	<0.001
Choking	16%	9%	0.007
Abdominal pain	11%	7%	0.056
Limited variety	61%	66%	0.133
Limited volume	35%	36%	0.936
Feeding refusal	9%	9%	0.863
Coughing	15%	8%	0.004
Regurgitation	0.3%	0.9%	0.296
Wheezing	0.6%	0.3%	0.588
Non-food	0.3%	0.6%	0.538

Esophagogastroduodenoscopy (EGD).

**Table 2:** Frequencies of EGD biopsy findings.

Pathology	Total Patients (%)
Esophagitis	132 (40)
Reflux (1–14 eosinophils/hpf)	96 (72.7)
EoE (≥ 15/hpf)	36 (27.3)
Gastritis	111 (33.6)
Chronic	67 (60.4)
Acute	3 (2.7)
Eosinophilic	35 (31.5)
*H. pylori*	6 (5.4)
Duodenitis	50 (15.2)
Chronic	10 (20)
Acute	5 (10)
Eosinophilic	35 (70)

hpf = high power field (400×); EoE = eosinophilic esophagitis.

**Table 3:** Frequencies of biopsy results in patients with and without a feeding tube.

Result	Feeding Tube	No Feeding Tube	*p*-Value
Esophagitis	37%	41%	0.501
Gastritis	35%	33%	0.802
Duodenitis	21%	13%	0.058

**Table 4:** Symptom frequencies of patients with and without esophagitis.

Symptom	Esophagitis	No Esophagitis	*p*-Value
Dysphagia	24%	23%	0.750
Gagging	35%	32%	0.634
Retching	0%	0.5%	1.000
Vomiting	35%	37%	0.708
Choking	18%	15%	0.391
Abdominal pain	11%	11%	1.000
Limited variety	67%	57%	0.066
Limited volume	37%	34%	0.605
Feeding refusal	11%	7%	0.177
Coughing	15%	15%	0.899
Regurgitation	0%	0.5%	1.000
Wheezing	0%	1%	0.519
Non-food	0%	0.5%	1.000

**Table 5:** Symptom frequencies of patients with and without gastritis.

Symptom	Gastritis	No Gastritis	*p*-Value
Dysphagia	22%	24%	0.601
Gagging	26%	37%	0.048
Retching	0%	0.5%	1.000
Vomiting	31%	39%	0.144
Choking	15%	16%	0.793
Abdominal pain	7%	10%	0.399
Limited variety	63%	59%	0.515
Limited volume	36%	35%	0.875
Feeding refusal	4%	11%	0.022
Coughing	16%	14%	0.619
Regurgitation	0.9%	0%	0.336
Wheezing	0.9%	0.5%	1.000
Non-food	0%	0.5%	1.000

**Table 6:** Symptom frequencies of patients with and without duodenitis.

Symptom	Duodenitis	No Duodenitis	*p*-Value
Dysphagia	20%	24%	0.545
Gagging	40%	32%	0.278
Retching	0%	0.4%	1.000
Vomiting	32%	37%	0.516
Choking	12%	17%	0.396
Abdominal pain	4%	12%	0.134
Limited variety	54%	62%	0.299
Limited volume	36%	36%	0.930
Feeding refusal	16%	8%	0.051
Coughing	22%	14%	0.123
Regurgitation	0%	0.4%	1.000
Wheezing	0%	0.7%	1.000
Non-food	2%	0%	0.152
